# Síntesis de evidencia y recomendaciones: guía de práctica clínica para el manejo de la retinopatía de la prematuridad

**DOI:** 10.26633/RPSP.2021.138

**Published:** 2021-12-01

**Authors:** Organización Panamericana de la Salud

**Affiliations:** 1 Organización Panamericana de la Salud Washington, D.C. Estados Unidos de América Organización Panamericana de la Salud, Washington, D.C., Estados Unidos de América

**Keywords:** Neonatología, prematuridad, prevención, retinopatía de la prematuridad, medicina basada en evidencia, terapéutica, Américas, Neonatology, prematurity, neonatal, prevention, retinopathy of prematurity, evidence-based medicine, therapeutics, Americas, Neonatologia, prematuridade, prevenção, retinopatia da prematuridade, medicina baseada em evidências, terapêutica, Américas

## Abstract

**Introducción.:**

La retinopatía del prematuro (ROP) es una enfermedad prevenible potencialmente grave que se puede presentar en hasta el 34% de los recién nacidos de pretérmino. La Organización Mundial de la Salud incluye el manejo de esta entidad dentro de sus políticas prioritarias para reducir la prevalencia de ceguera prevenible.

**Objetivos.:**

Sintetizar las recomendaciones incluidas en la *Guía de práctica clínica para el manejo de la retinopatía de la prematuridad*, publicada por la Organización Panamericana de la Salud, con el fin de presentar recomendaciones para la prevención, diagnóstico, tratamiento y seguimiento de recién nacidos con retinopatía del prematuro.

**Métodos.:**

Se llevó a cabo una síntesis de la guía y sus recomendaciones. Además, se realizó una búsqueda sistemática en Pubmed, Lilacs, Health Systems Evidence, Epistemonikos y literatura gris de estudios desarrollados en la Región de las Américas con el fin de identificar barreras, facilitadores y estrategias de implementación.

**Resultados.:**

Se formularon 30 recomendaciones y 14 puntos de buena práctica que aplican a los recién nacidos prematuros menores de 32 semanas de edad gestacional y/o menos de 1500 g de peso al nacer que hayan requerido oxígeno o presenten otros factores de riesgo para presentar ROP. Se identificaron barreras de acceso relacionadas con la disponibilidad de recurso humano, insumos y conocimiento de la guía para la implementación de las recomendaciones.

**Conclusiones.:**

Las recomendaciones formuladas buscan proveer estrategias para la prevención, el diagnóstico y el tratamiento de ROP para los recién nacidos prematuros en América Latina y el Caribe, así como consideraciones para su implementación.

## Abstract (en)

**Introduction.:**

Retinopathy of prematurity (ROP) is a potentially serious preventable disease that can present in up to 34% of preterm newborns. The World Health Organization includes the management of this entity among its priority policies for reducing the prevalence of preventable blindness.

**Objectives.:**

Synthesize the recommendations contained in *Clinical Practice Guidelines for the Management of Retinopathy of Prematurity*, a publication of the Pan American Health Organization, with a view to producing recommendations for the prevention, diagnosis, treatment, and monitoring of newborns with retinopathy of prematurity.

**Methods.:**

The guidelines and recommendations were synthesized and a systematic search was conducted in PubMed, Lilacs, Health Systems Evidence, Epistemonikos, and gray literature for studies carried out in the Region of the Americas with a view to identifying barriers, facilitators, and implementation strategies.

**Results.:**

A total of 30 recommendations and 14 good practices were formulated for application to premature newborns under 32 weeks of gestational age and/or with a birthweight of less than 1500 g who have required oxygen or present other risk factors for ROP. Barriers to implementation were lack of available human resources, supplies and equipment, and knowledge about guidelines for implementation of the recommendations.

**Conclusions.:**

The formulated recommendations aim to provide strategies for the prevention, diagnosis, and treatment of ROP in premature newborns and include suggestions for their implementation in Latin America and the Caribbean.

## RESUMO

**Introdução.:**

A retinopatia da prematuridade (ROP) é uma doença prevenível potencialmente grave que pode se apresentar em até 34% dos recém-nascidos prematuros. A Organização Mundial da Saúde inclui o manejo dessa condição em suas políticas prioritárias para reduzir a prevalência da cegueira prevenível.

**Objetivos.:**

Sintetizar as recomendações incluídas no *Guia de prática clínica para o manejo da retinopatia da prematuridade*, publicado pela Organização Pan-Americana da Saúde, com o fim de apresentar recomendações para a prevenção, diagnóstico, tratamento e seguimento de recém-nascidos com retinopatia da prematuridade.

**Métodos.:**

Foi realizada uma síntese do guia e de suas recomendações. Além disso, foi feita uma busca sistemática nas bases Pubmed, Lilacs, Health Systems Evidence e Epistemonikos, e na literatura cinzenta de estudos conduzidos na Região das Américas para identificar barreiras, facilitadores e estratégias de implementação.

**Resultados.:**

Foram formuladas 30 recomendações e 14 pontos de boas práticas que se aplicam aos recém-nascidos prematuros com menos de 32 semanas de idade gestacional e/ou menos de 1.500g de peso ao nascer, que precisaram de oxigênio ou apresentaram outros fatores de risco para a ROP. Foram identificadas barreiras de acesso relacionadas com a disponibilidade de recurso humano, insumos e conhecimento do guia para a implementação das recomendações.

**Conclusões.:**

As recomendações formuladas buscam fornecer estratégias para a prevenção, o diagnóstico e o tratamento da ROP em recém-nascidos prematuros na América Latina e no Caribe, bem como considerações para sua implementação.

La retinopatía del prematuro (ROP) es una enfermedad potencialmente grave que se presenta en recién nacidos prematuros, la cual afecta los vasos sanguíneos de la retina en desarrollo. La ROP se presenta como resultado de la aparición de cortocircuitos vasculares, la neovascularización; en sus formas más graves, ocurre tracción y desprendimiento de retina (1). La ROP solo aparece en los recién nacidos prematuros con retina inmadura y vascularización incompleta (2). La principal causa asociada a la ROP es la administración de oxígeno mal controlada en las salas de parto o unidades de cuidados intensivos neonatales (UCIN) de los recién nacidos de pretérmino (3).

Se ha observado la aparición de ROP aún en ausencia de oxígeno complementario (4), asociada con cardiopatías congénitas cianosantes y anencefalia (5). Otros factores de riesgo que se han asociado con la ROP incluyen la apnea; el uso de nutrición parenteral prolongada; la cantidad de transfusiones de sangre; los episodios de hipoxemia, hipercarbia e hipocarbia; y la septicemia (6). También influyen en la aparición de ROP el número de horas en ventilador, la administración de xantinas y la presencia de sangrado de la madre (7). La ROP es una de las principales patologías causantes de ceguera prevenible en niños (8). Puede afectar hasta a 34% de los prematuros con menos de 1500 gramos de peso al nacer, de los cuales 6 a 27% requerirán tratamiento (9). Una revisión sistemática identificó las prevalencias poblacionales de ROP en América Latina:

- Argentina (2010): 26,2% de todos los niños prematuros.
- Bolivia (Estado Plurinacional de) (2002): 14,3%.
- Brasil (2010): 9,3%.
- Chile (2004): 12,3.
- Cuba (2010): 5,1%.
- Guatemala (2010): 13%.
- Nicaragua (2004): 23,8%.
- Perú (2007): 19,1% (10), notifican una prevalencia de 9,4% (11).
- Colombia (2016): notifica una prevalencia de 3,19% por cada 10 000 nacidos vivos (12).

Teniendo en cuenta estos factores, la Organización Mundial de la Salud (OMS) mediante su programa Visión 2020, incluye el manejo de esta entidad dentro de sus políticas prioritarias para mejorar la salud visual y reducir la prevalencia de ceguera prevenible (13). Debido a estas consideraciones, se vuelve necesario establecer los lineamientos de prevención, diagnóstico y tratamiento de la ROP en la Región de las Américas.

El objetivo de este trabajo es presentar una síntesis de evidencia de las recomendaciones presentadas en la *Guía de práctica clínica para el manejo de la retinopatía de la prematuridad* (14) publicada por la Organización Panamericana de la Salud en el 2018, y presentar aspectos relevantes para su implementación en la Región.

## MÉTODOS

### Objetivos y población diana considerada en la guía

La *Guía de práctica clínica para el manejo de la retinopatía de la prematuridad* (14) se desarrolló con los siguientes objetivos: 1) identificar de forma temprana y prevenir los factores de riesgo causantes de la ROP en recién nacidos de pretérmino durante su estancia en la UCIN; y 2) presentar las estrategias disponibles para el diagnóstico, el tratamiento y el seguimiento de recién nacidos con ROP en América Latina. La población diana está constituida por: 1) recién nacidos prematuros menores de 32 semanas de edad gestacional y/o menos de 1500 g de peso al nacer; y 2) recién nacidos prematuros con edades gestacionales comprendidas entre las 33 y las 36 semanas inclusive, de cualquier peso al nacer, que hayan requerido oxígeno o presenten otros factores de riesgo para presentar ROP en algún momento entre su nacimiento y el egreso hospitalario.

### Metodología de elaboración de la guía

La guía siguió el método de desarrollo rápido de las guías elaboradas con el sistema GRADE propuesto por la OPS: *Directriz para el fortalecimiento de los programas nacionales de guías informadas por la evidencia. Una herramienta para la adaptación e implementación de guías en las Américas* (15) y del *Manual para elaborar guías de la Organización Mundial de la Salud* (16). En general, se conformó un grupo desarrollador multidisciplinario compuesto por expertos temáticos, epidemiólogos, sanitaristas y usuarios. Se identificó una guía pasible de ser adaptada. Sin embargo, la mayoría de las preguntas de la guía se respondieron con base en revisiones sistemáticas de la literatura. Se realizaron búsquedas hasta enero del 2017 en bases de datos electrónicas (Pubmed, EMBASE, Cochrane y Epistemonikos), búsqueda manual y literatura gris. Luego, se elaboraron la síntesis y los perfiles de evidencia utilizando el enfoque GRADE (17). Todos los participantes del panel y del grupo desarrollador firmaron un formulario de conflicto de intereses, los cuales fueron analizados por la coordinación de la guía. El enfoque GRADE permite formular las recomendaciones considerando la calidad de la evidencia, el balance entre el riesgo y el beneficio, los valores y preferencias de los pacientes, la aplicabilidad, los costos y el contexto global de implementación. Los detalles metodológicos y la evidencia que apoya las recomendaciones están disponibles en la guía que sirvió de base para este trabajo (14).

### Alcance y usuarios de la guía

Las recomendaciones informadas en la evidencia incluidas en la guía que sirvió de base para este trabajo versan sobre la prevención, el diagnóstico, el tratamiento y el seguimiento de la ROP, respetando el ámbito local o regional, sin importar el sistema de salud. Las recomendaciones están dirigidas a todos los oftalmólogos pediatras, neonatólogos, retinólogos, pediatras, enfermeras y profesionales que laboran en unidades neonatales con el fin de efectuar la prevención y manejo de retinopatía del prematuro. La guía pretende ser usada por tomadores de decisiones y miembros de entidades gubernamentales con el fin de facilitar el proceso de implementación. Solo incluye recomendaciones de cuidado de niños prematuros asociadas a la ROP. Para el cuidado general de los niños prematuros, se recomienda que cada país implemente sus programas, políticas y guías nacionales informadas en la evidencia sobre el tema (14).

## CÓMO USAR ESTA GUÍA

Para cada pregunta clínica se presenta un grupo de recomendaciones y buenas prácticas para el manejo de la retinopatía de la prematuridad.

En los cuadros 1 y 2 se presentan el nivel de calidad de la evidencia y la fuerza de la recomendación según el sistema GRADE, respectivamente (17).

## METODOLOGÍA DE DESARROLLO DE LA SÍNTESIS DE EVIDENCIA

La *Guía de práctica clínica para el manejo de la retinopatía de la prematuridad* (14), una guía de práctica desarrollada por la OPS, aborda esta causa de ceguera prevenible en recién nacidos prematuros de la Región de las Américas y cuya prevención es prioritaria para el avanzar hacia el logro de los Objetivos de Desarrollo Sostenibles (ODS). Se sintetizó la información de la guía relacionada con la metodología, el alcance, los objetivos, el resumen de las recomendaciones y la calidad de la evidencia mediante un formato predeterminado. Se utilizó la estrategia de búsqueda de la guía y filtros para identificar estudios sobre consideraciones de implementación (18), para detectar revisiones sistemáticas que abordaran aspectos de implementación (barreras, facilitadores, estrategias de implementación e indicadores). La estrategia incluyó los términos *adoption, uptake, utilization; taken implementation, dissemination, evidence-based treatment, barriers*. La búsqueda se realizó en Pubmed, Lilacs, Health Systems Evidence y Epistemonikos hasta mayo del 2020. Asimismo, se revisaron los estudios primarios e informes técnicos desarrollados en la Región; también se incluyeron las guías regionales y otros documentos de la OPS. No se evaluó la calidad de la evidencia incluida. Se seleccionaron revisiones sistemáticas y estudios primarios con el objetivo de identificar las consideraciones de implementación de las recomendaciones de la guía. Estas se organizaron de acuerdo con el tipo de barrera (factores humanos, preferencia de los pacientes, conocimiento de la guía, recursos y acceso). Para las barreras identificadas se seleccionaron los facilitadores y las estrategias de implementación más efectivas considerando el contexto de la Región. A partir de la guía, se identificaron indicadores de proceso y de resultado de su implementación. Por último, un grupo interdisciplinario de metodólogos y expertos temáticos de la OPS revisó los aspectos de implementación.

## RESULTADOS Y DISCUSIÓN

En el cuadro 3 se presentan las recomendaciones y los puntos de buena práctica que brindan orientación sobre el manejo de la ROP (14). Para cada pregunta clínica, se presenta en la guía el proceso de toma de decisiones para formular las recomendaciones de acuerdo con el enfoque GRADE (17). Las recomendaciones marcadas con * son clave para el proceso de implementación.

**CUADRO 1.** Nivel de calidad de la evidencia según el sistema GRADE

Nivel de evidencia	Significado
**Alta ⊕⊕⊕⊕**	Es muy poco probable que nuevos estudios cambien la confianza que se tiene en el resultado estimado.
**Moderada ⊕⊕⊕Ο**	Es probable que nuevos estudios tengan un impacto importante en la confianza que se tiene en el resultado estimado y que estos puedan modificar el resultado.
**Baja ⊕⊕ΟΟ**	Es muy probable que nuevos estudios tengan un impacto importante en la confianza que se tiene en el resultado estimado y que estos puedan modificar el resultado.
**Muy baja ⊕ΟΟΟ**	Cualquier resultado estimado es muy incierto.

**CUADRO 2.** Fuerza de la recomendación y su significado según el sistema GRADE

Fuerza de la recomendación	Significado
**Fuerte**	Las consecuencias deseables claramente sobrepasan las consecuencias indeseables. **SE RECOMIENDA HACERLO**
**Condicional**	Las consecuencias deseables probablemente sobrepasan las consecuencias indeseables. **SE SUGIERE HACERLO**

**CUADRO 3.** Treinta recomendaciones y catorce buenas prácticas sobre la guía para el manejo de retinopatía de la prematuridad en la Región de las Américas

Pregunta 1. ¿Cuáles son los factores de riesgo o protectores para la ocurrencia de retinopatía de la prematuridad (ROP)?	
Grado de recomendación	Recomendaciones de la guía
Fuerte a favor	Se recomienda el uso de la alimentación enteral utilizando leche humana y calostro en recién nacidos prematuros por su efecto protector en la incidencia de ROP. Calidad de la evidencia: muy baja ⊕ΟΟΟ
Condicional a favor	Se recomienda la administración de lactoferrina oral por su efecto en la reducción de la incidencia de ROP en los países que esté disponible. Calidad de la evidencia: muy baja ⊕ΟΟΟ
Condicional a favor	Se sugiere crear un sistema de alarma de riesgo de ROP en las unidades de cuidado intensivo neonatal que evalué la edad gestacional, ganancia de peso y el peso al nacimiento con el fin de determinar el riesgo de ROP.
Condicional a favor	Se sugiere suplementación con vitamina A, vitamina E o inositol a los recién nacidos prematuros por su efecto en la disminución de ROP. Calidad de la evidencia: muy baja ⊕ΟΟΟ
Fuerte en contra	No se recomienda el uso de eritropoyetina debido a que aumenta la incidencia de ROP grave. Calidad de la evidencia: baja ⊕⊕ΟΟ
Fuerte a favor	En recién nacidos prematuros en sala de nacimientos, se recomienda iniciar la reanimación empleando ventilación a presión positiva con niveles bajos de oxígeno (entre 21% y 30%), además de monitorear la saturación de oxígeno en forma constante.* Calidad de la evidencia: baja ⊕⊕ΟΟ
Fuerte a favor	En las salas de nacimiento, se recomienda mantener los siguientes rangos de saturación en los neonatos pretérmino con riesgo de desarrollar ROP: 3 minutos: 70-75% 5 minutos: 80-85% 10 minutos: 85-95% Calidad de la evidencia: baja ⊕⊕ΟΟ
Fuerte a favor	Se recomienda ajustar los niveles de oxígeno (incremento o reducción) cada 90 segundos, tomando como referencia los parámetros esperados a los 3, 5 y 10 minutos. Calidad de la evidencia: baja ⊕⊕ΟΟ
Condicional a favor	Se sugiere monitorizar permanentemente la saturación de oxígeno, utilizando un oxímetro de pulso, mantener la saturación de oxígeno entre 89% y 94% y colocar la alarma de saturación mínima en 88% y la de saturación máxima en 95% en todos los recién nacidos prematuros a los que se esté administrando oxígeno. Calidad de la evidencia: moderada ⊕⊕⊕Ο
Punto de buena práctica	Se sugiere que todas las UCIN cuenten con mezcladores de aire comprimido y oxígeno, y oxímetros ambientales para controlar periódicamente la FiO_2_ (fracción inspirada de oxígeno), especialmente cuando se presentan discordancias entre la mezcla indicada y las saturaciones logradas.
Punto de buena práctica	Al realizar la higiene bronquial a través del tubo endotraqueal, se debe efectuar con sistema de aspiración cerrada. Comentario: Esto se realiza para que el niño reciba la misma concentración de oxígeno que estaba recibiendo y, para evitar episodios de hipoxia o hiperoxia, considerar otras estrategias (aumento de la presión inspiratoria máxima y la frecuencia respiratoria), en lugar de “preoxigenar” al niño aumentando la FiO_2_.
Punto de buena práctica	Se sugiere contar con flujómetros de bajo flujo (1 a 3 litros/minuto) y comunes de 15 litros/minuto.
	Los de bajo flujo deberían utilizarse cuando se usan cánulas nasales. Cuando se utiliza la cámara cefálica, el flujo debería ser de 8 a 10 litros/minuto con un mínimo de 5 litros en los pacientes más pequeños. Cuando se usa el sistema de presión positiva continua en la vía aérea, se recomienda utilizar un flujo más bajo para alcanzar la presión de final de espiración positiva deseada.
**Pregunta 2. ¿cuál es la utilidad y condiciones del tamizaje de retinopatía de la prematuridad en recién nacidos prematuros?**	
Fuerte a favor	Se recomienda emplear la edad gestacional y el peso al nacer, independientemente de la restricción del crecimiento extrauterino, como criterios para el tamizaje de ROP y no emplear solo la ganancia de peso en las primeras semanas de vida como criterio para el tamizaje de ROP. Calidad de la evidencia: muy baja ⊕ΟΟΟ
Fuerte a favor	Se recomienda realizar tamizaje para detección de ROP en todo recién nacido con peso al nacer de <2000 g y/o de 36 semanas o menos de EG con cualquier peso, que presente al menos una de las situaciones identificadas como factores de riesgo de ROP*. Calidad de la evidencia: muy baja ⊕ΟΟΟ
Fuerte a favor	Se recomienda que el primer examen para el tamizaje de ROP se realice antes del egreso de la unidad de cuidado neonatal: Recién nacidos al egreso con menos de 27 semanas, la valoración oftalmológica se realice al cumplir 30 semanas. Los recién nacidos con más de 27 semanas al egreso, la valoración oftalmológica se realice a la 4 semana de vida. Calidad de la evidencia: muy baja ⊕ΟΟΟ (Recomendación de consenso de expertos)
Fuerte a favor	El esquema de seguimiento para el tamizaje de ROP, deberá realizarse de acuerdo con el siguiente cuadro: **Figure:** 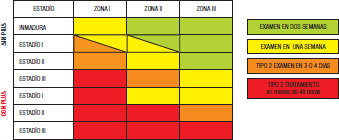 ***Fuente***: Ministerio de Salud y Protección Social de Colombia. Estrategia de atención de la primera infancia: de cero a siempre. Bogotá: MPSP; 2013.
	Calidad de la evidencia: Muy Baja ⊕ΟΟΟ (Recomendación por consenso de expertos)
Punto de buena práctica	Se sugiere registrar adecuadamente los resultados de cada examen oftalmológico, detallando la zona, estadio y extensión en términos de una “carátula para reloj” de cualquier tipo de ROP y la presencia de enfermedad preplus o enfermedad plus.
Fuerte a favor	Se recomienda suspender los exámenes oftalmológicos en recién nacidos sin ROP cuando la vascularización de la retina se ha extendido a la zona III y no antes de la semana 37. Calidad de la evidencia: muy baja ⊕ΟΟΟ
Condicional a favor	Se sugiere que, en presencia de ROP, el tamizaje de enfermedad activa sea discontinuada cuando algunas de las siguientes características estén presentes en por lo menos dos exámenes sucesivos: Falta de aumento de la gravedad de la enfermedad. Regresión completa de la ROP. Resolución parcial que progresa a completa. Cambio de color del reborde de rosa salmón a blanco. Transgresión de los vasos a través de la línea demarcatoria. Comienzo del proceso de reemplazo de las lesiones de ROP activa por parte de tejido cicatricial. 45 semanas cumplidas. Calidad de la evidencia: muy baja ⊕ΟΟΟ
Punto de buena práctica	Una vez finalizado el tamizaje para ROP potencialmente tratable, podrían continuarse los exámenes oftálmicos en caso de que el especialista considere que existe una probabilidad de identificar secuelas oftálmicas significativas pasibles de ser tratadas.
**Pregunta 3. ¿Cuál es la técnica de tamizaje de retinopatía de la prematuridad a utilizar en recién nacidos prematuros?**	
Fuerte a favor	Previo al examen de tamizaje para ROP, para dilatar la pupila del recién nacido se recomienda instilar una gota de una solución combinada de fenilefrina al 2,5% con tropicamida al 0,5% en cada ojo, en 2 o 3 dosis, con 15 minutos de diferencia entre una aplicación y otra.* Comentario: cada país puede adaptar la concentración según la disponibilidad y presentación del medicamento, sin que la concentración de epinefrina exceda el 2,5%. Calidad de la evidencia: baja ⊕⊕ΟΟ
Fuerte a favor	Se recomienda utilizar la menor cantidad posible y dosis de gotas midriáticas para dilatar las pupilas, monitorizando durante el proceso la frecuencia cardíaca, la respiratoria y la presión arterial del recién nacidos. Calidad de la evidencia: baja ⊕⊕ΟΟ
Punto de buena práctica	La aplicación de las gotas para dilatar la pupila debe hacerse por lo menos una hora antes del examen.
Fuerte a favor	Se recomienda utilizar gotas anestésicas antes del examen oftalmológico (p. ej., hidroclorato de proparacaína al 0,5%, 1 a 2 gotas, 30 a 60 segundos antes) si se utilizará separador (espéculo) palpebral o indentación escleral. Calidad de la evidencia: baja ⊕⊕ΟΟ
Fuerte a favor	Se recomienda emplear técnicas para disminuir el estrés y el dolor del recién nacido durante el examen oftalmológico para el tamizaje de ROP, tales como: administración de una solución de sacarosa, apoyarlo en el regazo materno, envolverlo con una sábana o el uso de un chupete. Calidad de la evidencia: baja ⊕ΟΟΟ
Punto de buena práctica Fuerte a favor	El tiempo para el examen oftalmológico para el tamizaje de ROP debe ser lo más corto posible, y se deben tomar las precauciones necesarias con el fin de resolver con prontitud y eficiencia cualquier situación de riesgo que se pudiera presentar como efectos en la presión arterial, la frecuencia cardíaca y la función respiratoria del recién nacido. Se recomienda emplear oftalmoscopia binocular indirecta (OBI) para realizar el tamizaje de ROP. Calidad de la evidencia: muy baja ⊕ΟΟΟ
Condicional a favor	Se sugiere utilizar sistemas de obtención de imágenes digitales cuando no se cuenta con oftalmólogos entrenados en el diagnóstico de esta patología, ya que permitiría transferir las imágenes a centros especializados de diagnóstico que cuenten con especialistas entrenados. Calidad de la evidencia: muy baja ⊕ΟΟΟ
Punto de buena practica	Durante el procedimiento, monitorear los niveles de tensión arterial, frecuencia cardíaca y saturación de O2, ya que pueden descender durante su realización. Calidad de la evidencia: muy baja ⊕ΟΟΟ
Fuerte a favor	Se recomienda que los oftalmólogos pediatras o retinólogos realicen el tamizaje en las unidades de cuidado neonatales. Calidad de la evidencia: muy baja ⊕ΟΟΟ
Condicional a favor	Se sugiere emplear el espéculo palpebral y el indentador escleral estériles para visualizar las regiones periféricas de la retina. Calidad de la evidencia: muy baja ⊕ΟΟΟ
**Pregunta 4. ¿Cuál son las indicaciones de tratamiento de recién nacidos diagnosticados con retinopatía de la prematuridad?**	
Fuerte a favor	Se recomienda que el tratamiento de ROP se realice en caso de presentarse alguna de las siguientes situaciones: Zona I: cualquier estadio de ROP con enfermedad plus. Zona II: estadio 3, sin enfermedad plus. Zona II: estadio 2, con enfermedad plus. Zona III: estadio 3 con enfermedad plus. Calidad de la evidencia: muy baja ⊕ΟΟΟ
Fuerte a favor	Se recomienda iniciar tratamiento dentro de las primeras 48 horas del diagnóstico a los niños con ROP agresiva posterior. En el resto de los casos, se sugiere tratarlos dentro de las 72 horas de diagnosticada.* Calidad de la evidencia: muy baja ⊕ΟΟΟ
Punto de buena práctica	Se sugiere que el oftalmólogo tratante explique a los padres la necesidad de tratamiento y obtener consentimiento informado antes de realizar el procedimiento.
Punto de buena práctica	Se sugiere que los niños que requieran tratamiento luego de haber sido dados de alta de su internación sean reingresados a una UCIN (o, en su defecto, una unidad de cuidado intensivo pediátrica).
Fuerte a favor	Se recomienda la terapia con láser diodo transpupilar como primera línea de tratamiento de recién nacidos con ROP. Calidad de la evidencia: baja ⊕⊕ΟΟ
Punto de buena práctica	Se sugiere que los recién nacidos con ROP sean tratados en la misma UCIN con sedación y analgesia.
	Puede realizarse el tratamiento en un quirófano con anestesia general, pero esto demora más tiempo y requiere un anestesiólogo con experiencia en pediatría, además del control del médico neonatólogo o pediatra y enfermera entrenada.
Punto de buena práctica	Se sugiere no utilizar la anestesia tópica como único medio para proveer analgesia en el tratamiento de la ROP.
Condicional a favor	Se sugiere considerar el uso de los medicamentos antagonistas del factor de crecimiento vascular endotelial (anti-VEGF, por su sigla en inglés) cuando no se tenga disponible el tratamiento quirúrgico de primera línea, y en los siguientes casos: Falla del tratamiento con láser. Cuando no es posible realizar el tratamiento con láser porque el niño está en condiciones críticas para tolerar el tratamiento o no es posible la visualización de la retina para realizar el tratamiento con láser o crioterapia. En recién nacidos con ROP agresiva posterior. En recién nacidos con ROP de tipo 1 en la zona I. Calidad de la evidencia: muy baja ⊕ΟΟΟ
Punto de buena práctica	Se debe explicar a los padres los beneficios y riesgos asociados al tratamiento, así como la falta de evidencia de eficacia y efectos a largo plazo y obtener la firma del consentimiento informado. Además, se debe confirmar el compromiso de seguir con el seguimiento del tratamiento.

Pregunta 5. ¿Cuáles son las indicaciones de seguimiento de recién nacidos tratados con retinopatía del prematuro?	
Condicional a favor	Se sugiere que al momento del egreso todo recién nacido con diagnóstico de ROP tratada o no tratada debe tener un plan de seguimientos periódicos con oftalmología, neonatología o pediatría y cita de seguimiento de recién nacido prematuro hasta que el criterio clínico lo considere pertinente.* Calidad de la evidencia: muy baja ⊕ΟΟΟ (Recomendación por consenso de expertos)
Fuerte a favor	Se recomienda que todo recién nacido tratado tenga un control posoperatorio durante la primera semana (4 a 8 días) para evaluar complicaciones o necesidad de retratamiento o tratamientos complementarios y según el criterio clínico. Calidad de la evidencia: muy baja ⊕ΟΟΟ (Recomendación por consenso de expertos)
Punto de buena práctica	El seguimiento debe hacerlo un oftalmólogo pediatra o retinólogo hasta verificar la vascularización de la retina. El seguimiento será a los 3, 6 y 12 meses para todos los niños prematuros, y después controles anuales.
Condicional a favor	Se sugiere realizar la derivación a estimulación visual temprana lo antes posible, desde los primeros meses de vida e incluso desde la internación neonatal a los recién nacidos que hayan presentado algún grado de ROP. Calidad de la evidencia: muy baja ⊕ΟΟΟ
Fuerte a favor	Se recomienda que los niños ciegos o con disminución visual sean integrados lo antes posible a la educación formal, ya sea común, especial o integrada de acuerdo a las características de su discapacidad, de su familia y de la oferta educativa de su comunidad. Calidad de la evidencia: muy baja ⊕ΟΟΟ (Recomendación por consenso de expertos)

**CUADRO 4.** Barreras, facilitadores y estrategias de implementación relacionados con el manejo de retinopatía de la prematuridad

Aspecto	Barreras	Facilitadores	Estrategias de implementación
Recurso humano	Dificultad de contar con especialistas en oftalmología pediátrica; oftalmólogos y optómetras capacitados	Proveedores de servicios de salud	Mejorar el entrenamiento de los profesionales en salud en las unidades de cuidado neonatal
	Falta de recurso humano para la adecuada realización de las pruebas de diagnóstico y el tratamiento en regiones aisladas	Instituciones educativas Entidades gubernamentales	Incremento de la oferta de profesionales de la salud para cumplir con las recomendaciones de manejo de ROP
Conocimiento de la guía de práctica clínica	Los profesionales de salud no conocen que existe una guía de manejo de ROP	Proveedores de servicios de salud	Socializar la guía a los profesionales de salud sobre dónde encontrar la guía en las instituciones
		Entidades gubernamentales	Alojar la guía en las páginas web de los repositorios nacionales de guías: páginas web de entidades gubernamentales, sociedades científicas y hospitales
		Sociedades científicas	Recordatorios en historias clínicas sistematizadas
			Difusión en revistas, boletines, aplicaciones móviles y páginas web
			Capacitaciones al personal de salud sobre las recomendaciones de la guía
Recursos	No todos los países cuentan con medicamentos en las dosis recomendadas lo cual puede afectar la salud de los recién nacidos	Entidades gubernamentales Proveedores de servicios de salud	Disponibilidad de nuevas tecnologías recomendadas en la mayoría de las instituciones de salud en el país Uso de tratamientos farmacológicos de acuerdo con la normatividad y recursos de cada país
Acceso	En áreas remotas se cuenta con poco acceso a especialistas y a tratamiento y seguimiento oportunos	UNICEF	Mejora en los sistemas de referencia y contrarreferencia regionales y nacionales
	Aspectos financieros del sistema de salud		Implementación de servicios de telemedicina
	Demora en las autorizaciones y trámites para recibir atención especializada		Formulación o fortalecimiento de las políticas de atención a niños prematuros Fortalecimiento de las redes de servicios neonatales y redes internacionales para la reducción de la ceguera prevenible

UNICEF: Fondo de las Naciones Unidas para la Infancia.

### Implementación

La guía recomienda que los siguientes actores apoyen la implementación de las recomendaciones (14): profesionales de la salud que atienden recién nacidos prematuros en las salas de nacimiento y en las UCIN (pediatras, neonatólogos, oftalmólogos, oftalmólogos pediatras, retinólogos, enfermeras especializadas); sociedades científicas; entes gubernamentales; organizaciones no gubernamentales; instituciones universitarias; administrativos de instituciones con unidades de cuidado neonatal; y actores clave de los sistemas de salud de cada país.

Dentro del proceso de implementación, es determinante identificar las posibles barreras, los facilitadores y las estrategias para mejorar la utilización de la guía. En el cuadro 4 se presentan algunos de estos elementos que pueden ser consideradas por los países (14,18,19).

En el recuadro 1 se sugieren los siguientes indicadores de proceso y resultado de la implementación de la guía (6).

**RECUADRO 1.** Indicadores de proceso y resultado en la implementación de la guía para el manejo de retinopatía de la prematuridad

Tasa de niños con ROP. Cobertura de tratamientos de ROP por unidad geográfica. Proporción de citas de habilitación y rehabilitación para niños con ROP por zona geográfica. Proporción de oftalmólogos por habitante. Número de niños prematuros tratados con los niveles de oxígeno recomendados.

## Conclusiones

La Organización Panamericana de la Salud pone a disposición de los gestores y del personal de la salud una síntesis sobre las recomendaciones informadas en la evidencia para la prevención, el diagnóstico, el tratamiento y el seguimiento de la retinopatía de la prematuridad. Asimismo, presenta aspectos a considerar, como algunas barreras para la implementación de las recomendaciones (p. ej., la falta de acceso a oftalmólogos capacitados, barreras de los sistemas de salud para referencias a especialistas y falta de conocimiento de la guía) y estrategias como el fortalecimiento de las políticas y programas de ceguera prevenible, creación de capacidad técnica local y apoyo en las redes nacionales e internacionales. Esperamos que esta síntesis favorezca la diseminación y el uso de las guías que elabora la OPS y contribuya a mejorar la calidad de la atención y la salud de los recién nacidos en la Región de las Américas

**Agradecimientos.** Por el apoyo para la elaboración de esta síntesis de evidencia: Dra. Ana Marcela Torres, Consultora del Departamento de Evidencia e Inteligencia para la Acción en Salud (EIH) de la OPS; Dr. Ludovic Reveiz, Asesor en Evidencia para la Salud Pública EIH/OPS; y Dr. Juan Carlos Silva, Asesor regional de la OPS Por el desarrollo de la guía, agradecemos a: Dr. Rodrigo Pardo, Del Instituto de Investigaciones Clínicas y Universidad Nacional de Colombia; Dr. Pablo Durán, Asesor regional de la OPS; Dr. Gabriel Lonngi Rojas, Neonatólogo, Coordinador académico de la División de Neonatología, Departamento de Pediatría, Universidad Nacional de Colombia; Dr. Juan Manuel Pardo, Oftalmólogo pediatra, Profesor del Departamento de Cirugía, Unidad de Oftalmología de la Facultad de Medicina, Universidad Nacional de Colombia; Dr. Andrés Galindo, Pediatra, Maestría en Epidemiologia clínica, Coordinador de investigación de la Sociedad Colombiana de Pediatría Regional Bogotá; Dra. María Teresa Vallejo; Dr. Carlos Grillo, Ginecólogo, Federación Colombiana de Ginecología; Dr. Jairo Amaya Profesor del Departamento de Ginecología y Obstetricia, Universidad Nacional de Colombia; Dra. Diana Fariña, Dra. Ana Quiroga y Dra. Patricia Fernández del Ministerio de Salud de la Nación de Argentina; Dr. Alejandro Colmenares y Dra. Clara Galvis del Hospital Militar Central de Bogotá; Dr. José María Solano de la Asociación Colombiana de Neonatología; Dra. Mónica Villa y Dr. Enrique Udaeta del Hospital Infantil de México; Dra. Tania Corpeño del Hospital Bertha Calderón y Ministerio de Salud de Nicaragua; Dra. María Josefa Castro del Hospital Dr. Miguel Pérez Carreño y Sociedad Venezolana de Puericultura y Pediatría; Dra. Martha Galán de la Nueva Clínica del Niño de La Plata de Argentina; Dra. Alexia Romanelli del Hospital del Niño de Bolivia; Dr. João Borges del Programa de prevención de ceguera por ROP de Brasil; Dra. Marcia Tartarella de la Sociedad de Oftalmopediatría Latinoamericana de Brasil; Dr. Alejandro Vaquez de Centromed de Chile; Dr. Diego Osssadon del Hospital de Niños Roberto del Río de Chile; Dr. Pedro Acevedo de la Fundación Oftalmológica Nacional de Colombia; Dra. Claudia Zuluaga de la Universidad del Valle de Colombia; Dra. Angela María Fernández de la Sociedad Colombiana de Oftalmología de Colombia; Dra. Marta Montenegro del Grupo de Investigación Visión de Unisanitas de Colombia; Dra. Ana Tabares del Hospital niños Carlos Saenz de Costa Rica; Dr. Alfonso Almeida de la Clínica Santa Lucía de Ecuador; Dr. Luis Orozco de Sociedad Mexicana de Oftalmología de México; Dr. Luis Gordillo del Programa de ROP de Perú; Dra. Clare Gilbert del London School of Hygiene and Tropical Medicine; Dr. Rolando Domínguez del Hospital Nacional de Niños Benjamín Bloom de El Salvador; Dr. Juan Ubiera de la Plaza de la Salud y Centro Láser de Republica Dominicana; Enf. Pamela Gallardo del Minsiterio de Salud de Chile; Enf Diana Hernández de la Universidad del Valle de Colombia.

## References

[1] Quinn GE, Gilbert C, Darlow BA, Zin A. Retinopathy of prematurity: an epidemic in the making. Chin Med J. 2010; 123(20):2929-2937. 21034609

[2] Blanco Teijeiro MJ. Retinopatía de la prematuridad. Archivos de la Sociedad Española de Oftalmología. 2006; 81:129-13. 10.4321/s0365-66912006000300001 16572353

[3] Fielder A, Blencowe H, O'Connor A, Gilbert C. (2015). Impact of retinopathy of prematurity on ocular structures and visual functions. Archives of Disease in Childhood - Fetal and Neonatal Edition. 2015: 100(2), F179-F184. 10.1136/archdischild-2014-306207 25336678

[4] Lucey JF, Dangman B. A reexamination of the role of oxygen in retrolental fibroplasia. Pediatrics. 1984; 73;1:82-96. 6419199

[5] Addison DJ, Font RL, Manschot WA. Proliferative retinopathy in anencephalic babies. Am J Ophthalmol 1972;74(5):967-976. 10.1016/0002-9394(72)91220-2 4644744

[6] Gunn TR, Easdown J, Outerbridge EW, Aranda JV. Risk factors in retrolental fibroplasia. Pediatrics. 1980; 65(6):1096. 6892851

[7] Hammer ME, Mullen PW, Ferguson JG, Pai S, Cosby C, Jackson KL. Logistic analysis of risk factors in acute retinopathy of prematurity. Am J Ophthalmol. 1986;102(1): 1-6. 10.1016/0002-9394(86)90200-x 3728608

[8] Marroquín G. Oftalmología pediátrica: guías de manejo. Colombia: Asociación Colombiana de Oftalmología Pediátrica y Estrabismo; 2016. Disponible en: https://acopecolombia.org/.

[9] Lomuto CC, Galina L, Brussa M, Quiroga A, Alda E, Benítez AM, et al. Epidemiología de la retinopatía del prematuro en servicios públicos de la Argentina durante 2008. Oftalmol Clin Exp 2010:3(4). 10.1590/S0325-00752010000100006 20204236

[10] Zimmermann-Paiz MA, Quiroga-Reyes CR. Catarata pediátrica en un país en vías de desarrollo: revisión retrospectiva de 328 casos. Arquivos Brasileiros de Oftalmología. 2011;74:163-165. 10.1590/s0004-27492011000300003 21915440

[11] Grupo ROP Argentina. Guía de práctica clínica para la prevención, diagnóstico y tratamiento de la retinopatía del prematuro (ROP). Buenos Aires: Ministerio de Salud; 2016. Disponible en: http://www.msal.gob.ar/images/stories/bes/graficos/0000000723cnt-guia-pract-clin-ROP-2015.pdf. Acceso en agosto de 2017.

[12] Ministerio de Salud y la Protección Social. Guía de práctica clínica: Detección de anomalías congénitas en el recién nacido. Guía N.° 03. Bogotá: Ministerio de Salud y la Protección Social; 2013. Disponible en: http://gpc.minsalud.gov.co/Documents/Guias-PDF-Recursos/Anomalias%20congenitas/GPC_Completa_Anom_Conge.pdf Fecha de acceso: agosto del 2017

[13] Organización Mundial de la Salud. Global initiative for the elimination of avoidable blindness: action plan 2006-2011. Vision 2020. The right to sight. Ginebra: OMS; 2007. Disponible en: https://apps.who.int/iris/handle/10665/43754. Acceso en junio del 2016.

[14] Organización Panamericana de la Salud. Guía de práctica clínica para el manejo de la retinopatía de la prematuridad. Washington, D.C.: OPS; 2018. Disponible en: http://iris.paho.org/xmlui/bitstream/handle/123456789/34948/9789275320020_spa.pdf?sequence=6&isAllowed=y.

[15] Organización Panamericana de la Salud. Directriz para el fortalecimiento de los programas nacionales de guías informadas por la evidencia. Una herramienta para la adaptación e implementación de guías en las américas. Washington, D.C.: OPS; 2018. Disponible en: http://iris.paho.org/xmlui/handle/123456789/49145. Acceso en junio de 2019.

[16] World Health Organization. Handbook for guideline development, 2° ed. Ginebra: OMS; 2014. Disponible en: https://www.who.int/publications/guidelines/handbook_2nd_ed.pdf?ua=1. Acceso en junio del 2016.

[17] Guyatt GH, Oxman AD, Kunz R, Atkins D, Brozek J, Vist G. et al. (2011). GRADE guidelines: 2. Framing the question and deciding on important outcomes. J Clin Epidemiol. 2011;64(4):395-400. 10.1016/j.jclinepi.2010.09.012 21194891

[18] Bain LC, Kristensen-Cabrera AI, Lee HC. A qualitative analysis of challenges and successes in retinopathy of prematurity screening. AJP reports. 2018;8(2):e128-e133. Disponible en: 10.1055/s-0038-1660519. PMC5995725 29896443

[19] Hariharan L, Gilbert CE, Quinn GE, Barg FK, Lomuto C, Quiroga A, et al. Reducing blindness from retinopathy of prematurity (ROP) in Argentina through collaboration, advocacy and policy implementation. Health Policy Plan. 20181;33(5):654-665. 10.1093/heapol/czy004 29668967

